# The New *G29A and G1222A of *HCRTR1*, 5-HTTLPR of *SLC6A4* Polymorphisms and Hypocretin-1, Serotonin Concentrations in Migraine Patients

**DOI:** 10.3389/fnmol.2018.00191

**Published:** 2018-06-05

**Authors:** Marta Kowalska, Magdalena Kapelusiak-Pielok, Teresa Grzelak, Ewa Wypasek, Wojciech Kozubski, Jolanta Dorszewska

**Affiliations:** ^1^Laboratory of Neurobiology, Department of Neurology, Poznan University of Medical Sciences, Poznan, Poland; ^2^Chair and Department of Neurology, Poznan University of Medical Sciences, Poznan, Poland; ^3^Department of Biology of Civilization-Linked Diseases, Poznan University of Medical Sciences, Poznan, Poland; ^4^Faculty of Medicine and Health Sciences, Andrzej Frycz Modrzewski Krakow University, Krakow, Poland; ^5^The John Paul II Hospital, Krakow, Poland; ^6^Institute of Cardiology, Jagiellonian University Medical College, Krakow, Poland

**Keywords:** 5-HTTLPR, *HCRTR1*, 5-HT, HCRT-1, migraine

## Abstract

Migraine is one of the most common primary headache disorders that affects 11% of the adult population. The disease is divided into two main clinical subtypes: migraine with aura (MA) and migraine without aura (MO). Both serotonergic and hypocretinergic systems are involved in the migraine pathomechanism. Polymorphisms in the serotonin transporter gene (*SLC6A4*) and the hypocretin receptor 1 gene (*HCRTR1*) may be risk factors for migraine development due to their ability to affect serotonin and hypocretin-1 (HCRT-1) concentrations. The aim of the study was to analyze, for the first time in the Polish population, the 5-HT transporter linked polymorphic region (5-HTTLPR) in *SLC6A4*, G1222A (rs2271933) and the never before studied *G29A (rs41263963) polymorphisms in the *HCRTR1* gene, as well as the 5-HT and hypocretin-1 plasma concentrations in migraine patients (MA, MO) and control subjects. The study included 123 patients that were diagnosed with migraine and 123 control subjects. Methods such as PCR, HRMA and sequencing were used for genotyping, while 5-HT was determined by HPLC/EC and hypocretin-1 by ELISA. No significant differences were observed in 5-HTTLPR frequencies. The A allele of *HCRTR1* G1222A occurred more often in MO, while the GA genotype of *HCRTR1* *G29A was more frequent among MA when compared to control group (*p* < 0.05). The mean age of migraine onset in individuals with *HCRTR1* *G29A was 18 years old for patients with MA and 26 years old for MO patients. The localization and type of *HCRTR1* polymorphisms (G1222A—missense variant in exon 7, *G29A−3′UTR variant) may predispose patients to the clinical subtype of migraine: MO or MA, respectively. In control subjects, the short allele of 5-HTTLPR tended to decrease the 5-HT concentration, while the A allele of *HCRTR1* G1222A decreased both 5-HT and hypocretin-1 levels. Serotonin concentrations differed in terms of clinical features of migraine. The relation between genotypes of 5-HTTLPR, *HCRTR1* G1222A, and 5-HT concentrations may bedisturbed in migraine. It seems that *HCRTR1* *G29A is more strongly associated with regulating the 5-HT in patients with MA than MO, and therefore may contribute to the early age of onset for migraine.

## Introduction

Migraine is a neurological disorder that occurs in two clinical subtypes: migraine with aura (MA) and without aura (MO). The disease affects 11% of the adult population worldwide and is three times more common among women than men (Stovner et al., 2007). According to the frequency of migraine attacks, migraine is divided into an episodic and chronic form. Chronic migraine, affecting 2% of the population, is characterized by headaches occurring for at least 15 days in a month (Menken et al., 2000). Migraine was classified by the World Health Organization (WHO) as the third most frequent disease, as well as the seventh cause of the decline in the quality of life, constituting a significant medical and social problem (Vos et al., 2013). The altered quality of life in those patients may also be explained by the fact, that migraine, especially the chronic form, is also comorbid with psychiatric disorders and suicidal behaviors (Friedman et al., 2018). Serafini et al. (2012) suggested that it may be a result of dysfunction in monoamine neurotransmission due to the possible association with gene variants.

The novel explanation of the migraine pathomechanism implies that the neurovascular origin in which the trigeminovascular system (TGVS) and cortical spreading depression (CSD) are activated. CSD is linked to migraine aura and activation of TGVS, also by CSD, is responsible for migraine pain. Activation of TGVS increases the level of pro-inflammatory mediators and causes sensitization of the pain-relevant regions of the brain. It was proposed that migraine may be a manifestation of the central biochemical dysfunction of pain perception (Sicuteri, 1976; Pietrobon and Striessnig, 2003). Pain perception is a complicated process mediated by the nociceptive system in which neurotransmitters such as serotonin (5-HT), hypocretins (HCRT), norepinephrine (NE) and gamma-aminobutyric acid (GABA) are involved (Sicuteri, 1976; Watanabe et al., 2005; Enna and McCarson, 2006).The trigeminovascular nociception involves the hypothalamus; a brain area that plays a role in primary headaches, among which migraine is the most common. The dysfunction of the hypothalamus is presented in the circadian rhythmicity of migraine and premonitory of attack, e.g., sleep deprivation, yawning, changes in appetite, in wakefulness, and in autonomic symptoms of the attack. It is proposed that HCRT may be responsible for migraines, mainly in the early phases of attack as hypocretinergic neurons originate from the hypothalamus and control sleep-wake cycle, food intake, pain modulation and regulate the autonomic system e.g., the serotoninergic system (Holland and Goadsby, 2007; Hoffmann et al., 2015).

Numerous studies indicate the role of the serotoninergic system in migraine pathophysiology (Hamel, 2007; Kowalska et al., 2016; Deen et al., 2017). During a migraine attack, plasma 5-HT concentration is higher, and its metabolite 5-hydroxyindoleacetic acid (5-HIAA) is lower than in attack-free periods. It is suggested that a migraine results from the chronically low 5-HT disposition (Ferrari et al., 1989; Ferrari and Saxena, 1993; Durham and Papapetropoulos, 2013). The cause of imbalance in 5-HT level may be polymorphism in 5-HT transporter (5-HTT) gene, *SLC6A4*. The 5-HTT linked polymorphic region (5-HTTLPR) is the insertion/deletion polymorphism located in the regulatory region of this gene. It occurs in two allelic forms: the short (S) and long variant (L), varying in length by 44 bp. Numerous studies have investigated the relationship between SS 5-HTTLPR genotype and migraine, but results are inconsistent, indicating its association either with migraine (Juhasz et al., 2003a), only with MA (Borroni et al., 2005; Marziniak et al., 2005), or MO (Gonda, 2007), or no association (Yılmaz et al., 2001; Szilagyi et al., 2006; Todt et al., 2006; Wieser et al., 2010). Additionally, Juhasz et al. (2003b) analyzed the correlation between 5-HTTLPR and 5-HT only in a small group of MO patients. The correlation between 5-HTTLPR and 5-HT in MA patients was not studied until now. Therefore, we decided to analyze, for the first time, the 5-HTTLPR in the Polish population of migraine sufferers.

The functions of serotonergic neurons are regulated by the hypocretinergic system, which consists of following neuropeptides: hypocretin-1 (HCRT-1) and hypocretin-2 (HCRT-2), and their receptors: HCRTR1, HCRTR2. The HCRTR1 receptor is selective for HCRT-1 (Holland and Goadsby, 2007). A good understanding of this pathway is important because the increased HCRT neural activity was observed in response to persistent pain and stress. HCRT-1 and HCRTR1 were found in the brain regions responsible for nociceptive processing: periaqueductal gray rostral ventral medulla, and in the lamina I of the spinal cord. Moreover, higher sensitivity to pain after peripheral inflammation and reduced stress-induced analgesia were found in HCRT-knockout mice as compared to control animals. Additionally, central administration of HCRT-1 in acute and persistent pain showed an analgesic effect. These findings suggest that HCRT neurons may inhibit pain transmission by themselves or by activating the descending pain inhibitory system (Ida et al., 2000; Bingham et al., 2001; Yamamoto et al., 2002; Cheng et al., 2003; Watanabe et al., 2005). The genetic study of Rainero et al. (2011b) suggested that non-synonymous G1222A polymorphism in *HCRTR1* gene is a risk factor for MO, but does not modify the clinical features of the disease. They did not focus on the possible association between the *HCRTR1* G1222A genotypes and HCRT plasma levels. Until now, the *HCRTR1* *G29A polymorphism was not analyzed previously in any disease. According to our preliminary study, performed on a group of 34 migraine patients, both 5-HTTLPR and *HCRTR1* G1222A polymorphisms may be associated with 5-HT and HCRT-1 concentrations (Kowalska et al., 2016). It was the first study in which the correlations between 5-HT and HCRT in combination with genetic analysis were analyzed. Thus, we continued the study on a bigger group of patients and analyzed the new *HCRTR1* *G29A polymorphism.

The aim of the study was to investigate the relationship between 5-HTTLPR and *HCRTR1* (G1222A, *G29A) polymorphisms and the concentration of 5-HT and HCRT-1 in plasma of MA, MO patients, and the control group.

We believe that these polymorphisms may be risk factors for MA and MO and may be associated with the lower concentration of 5-HT and HCRT-1.

## Materials and Methods

### Patients and Controls

The study was performed in 246 individuals, both migraine patients and control subjects.

Between 2015 and 2017, a total of 123 migraine patients (112 females, 11 males; mean age ± SD: 40 ± 14 years) were selected for the study from the Department of Neurology at Poznan University of Medical Sciences (PUMS) and the neurology clinic. The diagnosis of migraine was performed according to the ICHD-3 criteria (Headache Classification Committee of the International Headache Society (IHS), 2003), by an experienced neurologist. Forty-nine patients fulfilled the diagnostic criteria for MA and 74 for MO.

The control group consisted of 123, sex and age-matched healthy subjects (112 females, 11 males, mean age ± SD: 39 ± 14 years). These participants were recruited via advertisements throughout PUMS. All individuals (patients and controls) were from the Polish Caucasian population. None of them were suffering from psychiatric or any other neurological disorders.

The clinical features of migraine: frequency of attacks and duration of a headache, were scored on a scale of 1–3, designed by Laboratory of Neurobiology PUMS. For frequency of a headache: 1–less than one attack per month, 2–one or two per month, 3–more than two per month. For the duration of a headache: 1–less than 24 h, 2–from 24 h to 48 h, 3–more than 48 h.

This study was carried out in accordance with the recommendations of the national legislation and the Code of Ethical Principles for Medical Research Involving Human Subjects of the World Medical Association. The protocol was approved by the Local Bioethical Committee of PUMS (no. 931/14 with an extension no. 993/17). All subjects gave written informed consents in accordance with the Declaration of Helsinki.

The blood was collected in the headache-free period. All samples were blinded to avoid bias. Genotyping was performed in all patients (treated and untreated) and control subjects. Individuals treated with triptans, antiepileptic and antidepressant drugs were excluded from biochemical studies (7 MA and 5 MO patients).

### Genetic Analyses

Genomic DNA was extracted using Genomic Micro AX Blood Gravity isolation kit (A&A Biotechnology, Poland) and stored at −80°C.

Polymerase chain reaction (PCR) of 5-HTTLPR was carried out using the following primers: 5′-GGCGT TGCCGCTCTGAATGC-3′ (forward) and 5′-GAGGGACT GAGCTGGACAACCAC-3′ (reverse) in reaction mixtures with a total volume of 15 μl, containing 45 ng genomic DNA, 10 pM of each primer, 4.5 nM dNTP, 5% DMSO, 1.125 U Hi-Fi Taq polymerase and 10× buffer for Hi-Fi Taq polymerase (Novazym, Poland). Temperatures were 63°C for 30 s for annealing and 72°C for 60 s for the extension. The 5-HTTLPR genotypes were identified by electrophoresis in 2% agarose gel, S allele had 484 bp, while L had 528 bp.

High-resolution melting analysis (HRMA) was used to genotype missense single nucleotide polymorphism (SNP) G1222A (rs2271933) in the exon 7 and *G29A (rs41263963) SNP in 3′UTR region of the *HCRTR1* gene, using LightCycler 480 Real-Time PCR System (Roche, USA). At the beginning, when we started analyzing the G1222A polymorphism of* HCRTR1* gene, we found that some patients had another polymorphism: *G29A. Therefore, we designed new primers to analyze *G29A alone without the effect of the G1222A polymorphism on the melt curve. The primers for HRMA were designed using online Primer3Plus software based on the published genome sequence of the *HCRTR1* gene and had the following sequences: G1222A: 5′-TCTCTGAAGGCCCCTAGTCC-3′ (forward) and 5′-GACTGAAGCCACAGCCTTTC-3′ (reverse); *G29A: 5′-CTCACCAGCGTCACCACA-3′ (forward) and 5′-CTCACATCAACCCTGCTTAGG-3′ (reverse). The results of HRMA were confirmed by sequencing in the forward and reverse directions using the 3130xl Genetic Analyzer (Applied Biosystems HITACHI, USA) in the independent unit. The reads were aligned to the human reference genome with BioEdit Software (Tom Hall Ibis Biosciences, Canada) separately by two investigators. The intercalating dye used was EvaGreen, and the reaction mixture with a total volume of 10 μl consisted of 1× SsoFast™ EvaGreen^®^ Supermix (BioRad, USA), 250 nM of each primer and 15 or 30 ng genomic DNA (G1222A or *G29A). The PCR reaction was performed as follows: initial denaturation for the 30 s at 95°C, followed by 45 cycles at 95°C for 10 s and 62°C for 30 or 20 s (G122A or *G29A). After PCR, samples were heated to 95°C for 30 s and then the melting was performed from 60°C to 95°C at the rate of 0.2°C/3 s or 0.3°C/2 s (G1222A or *G29A).

### Biochemical Analyses

The plasma 5-HT concentration was determined by high-pressure liquid chromatography coupled with electrochemical detection (HPLC/EC). The 0.1 M acetate buffer, pH 5.0 was added to the plasma samples and 5-HT standards (Sigma-Aldrich, USA) in a ratio of 1:3 and mixed. The samples were deproteinized with 8 M HClO_4_ in a ratio of 10:1 and fed to a HPLC/EC system. The analysis was carried out on a Thermo Hypersil BDS C18 (250 mm × 4.6 mm × 5 μm) column (Germany), in isocratic conditions using a mobile phase of 10 mM phosphate buffer, pH 5.0 containing 10% methanol. The system was controlled, and data was collected and processed using Chromeleon software (Dionex, Germany).

The plasma concentration of HCRT-1 was evaluated using commercially available enzyme-linked immunoassay (ELISA) kit (Wuhan EIAlab Science Co., China) according to the manufacturer’s protocol (inter- and intra-assay coefficients of variability amounted: ≤8.5% and ≤6.7%, respectively).

### Statistics

The sample size was calculated with the acceptable level of alpha error α < 0.05 and the estimation error of 0.09 on the assumption that 11% of Wielkopolska Voivodeship population suffers from migraine. The sample size was fixed at 119 patients.

The analyses were performed for MA and MO patients both separately and combined. The Fisher’s exact test, Chi-square test and odds ratio (OR) with 95% confidence intervals (95% CI) were applied for analysis of the distribution of alleles and genotypes. Hardy-Weinberg equilibrium was verified for all the polymorphisms in MA, MO and control groups using Chi-square test. The normality of the data (5-HT, HCRT-1) distributions was checked using the Shapiro-Wilk test (if the sample size were 50 or less) and Kolgomorov-Smirnow (if the sample size were larger than 50). Analysis of the homogeneity of variance was carried out using the Levene test. The differences in 5-HT or HCRT-1 levels between groups with each genotype were tested by Kruskal-Wallis tests (in the case of three groups) and Mann-Whitney test (in the case of two groups). Correlations between 5-HT and HCRT-1 were tested using Spearman’s rank test. GraphPad (Instant) and Statistica 12.0 for Windows (StatSoft, USA) were used to evaluate the results of the study. Two-tailed *p* < 0.05 were considered statistically significant in the case of two groups. Considering the potential false positive rate incurred by multiple comparisons of several gene associations in controls and two patient groups, we applied the Bonferroni correction method to adjust the *p*-value.

## Results

The MA and MO groups differed regarding the duration of migraine history, mean number of attacks per month and duration of the attacks. The majority of MA patients suffer from migraine for more years, experience fewer attacks, which last shorter than migraine attacks in MO group (Table 1).

**Table 1:** Clinical description of the migraine patients.

Characteristic	MA	MO
Mean age at interview (years)	39 ± 14	40 ± 15
Duration of migraine history (years):		
<5	10.2%	11.1%
5–10	20.4%	61.1%
>10	69.4%	27.8%
Mean number of attacks per month:		
<1	33.3%	12.2%
1–2	47.9%	48.6%
>2	18.8%	39.2%
Mean duration of attacks (hours):		
<24	34.0%	16.7%
24–48	34.0%	52.8%
>48	32.0%	30.5%
Unilateral pain	93.5%	84.7%
Pulsating pain	73.3%	74.0%
Nausea	91.3%	90.4%
Vomiting	73.9%	52.1%
Photophobia	89.1%	91.9%
Phonophobia	77.8%	82.4%
Osmophobia	72.7%	68.1%

### Genetic Analyses

None of the analyzed polymorphisms showed significant deviation from Hardy-Weinberg equilibrium in either MA, MO or control subjects. In the control group, the minor allele frequency (MAF) for analyzed nucleotide variants was at least 3.3%. The power to detect a disease susceptibility locus was between 88% and 99%.

No statistical differences were found in frequencies of 5-HTTLPR (Table 2). However, the genotype and allele frequencies of the analyzed polymorphisms in *HCRTR1* varied between MA, MO patients, and healthy controls. The A allele of *HCRTR1* G1222A was more frequent in the MO group than in the control group (*p* = 0.0414, Chi-square test), while GA genotype of *HCRTR1* *G29A was more common in the MA group compared to the control group (*p* = 0.0453, Fisher’s exact test). The differences in *HCRTR1* G1222A and *G29A frequencies were not statistically significant when we considered MA and MO patients together.

**Table 2:** Genotype and allele frequencies of 5-HTT linked polymorphic region (5-HTTLPR) and hypocretin receptor 1 gene (*HCRTR1*) G1222A, *G29A polymorphisms.

	Controls	MA	MO	Migraine patients (MA+MO)
5-HTTLPR				
Genotypes:				
SS (%)	12 (9.7)	5 (10.2)	12 (16.2)	17 (13.8)
SL (%)	59 (48.0)	29 (59.2)	35 (47.3)	64 (52.0)
LL (%)	52 (42.3)	15 (30.6)	27 (36.5)	42 (34.2)
Alleles:
S (%)	83 (33.7)	39 (39.8)	59 (39.9)	98 (39.8)
L (%)	163 (66.3)	59 (60.2)	89 (60.1)	148 (60.2)
*HCRTR1* G1222A				
Genotypes:				
GG (%)	43 (35.0)	14 (28.6)	14 (18.9)*	28 (22.8)
GA (%)	58 (47.1)	24 (49.0)	43 (58.1)*	67 (54.4)
AA (%)	22 (17.9)	11 (22.4)	17 (23.0)	28 (22.8)
Alleles:				
G (%)	144 (58.5)	52 (53.1)	71 (48.0)	123 (50.0)
A (%)	102 (41.5)	46 (46.9)	77 (52.0)*	123 (50.0)
*HCRTR1* *G29A				
Genotypes:				
GG (%)	115 (93.5)	41 (83.7)*	65 (87.8)	106 (86.2)
GA (%)	8 (6.5)	8 (16.3)*	9 (12.2)	17 (13.8)
AA (%)	0 (0.0)	0 (0.0)	0 (0.0)	0 (0.0)
Alleles:				
G (%)	238 (96.7)	90 (91.8)	139 (93.9)	229 (93.1)
A (%)	8 (3.3)	8 (8.2)	9 (6.1)	17 (6.9)

MA, migraine with aura, MO, migraine without aura. *p < 0.05 compared to controls, Fisher’s exact test or Chi-square test, as appropriate.

None of the alleles were associated with increased migraine risk, although the A allele of *HCRTR1* G1222A (*p* = 0.0574) and the A allele of *HCRTR1* *G29A (*p* = 0.0647) approached the borderline of significance (Table 3). Interestingly, the majority of MA individuals with GA *HCRTR1* *G29A shared the same clinical feature: the early onset of the disease: migraine started before the age of 18. The mean age of onset of the disease in GA carriers of *HCRTR1* *G29A for MO was 26 years. None of the examined subjects had the AA genotype of *HCRTR1* *G29A.

**Table 3:** Genotypic and allelic distribution of 5-HTTLPR and *HCRTR1* G1222A, *G29A polymorphisms in migraine patients (MA+MO) compare to control subjects.

	Chi-square	p	OR	95% CI
5-HTTLPR				
Alleles:				
S vs. L	1.967	0.1608	1.30	0.90–1.88
Genotypes:				
SS vs. LL	1.725	0.1890	1.75	0.76–4.08
SL vs. LL	1.152	0.2830	1.34	0.78–2.30
SS vs. SL	0.409	0.5225	1.31	0.58–2.96
*HCRTR1* G1222A				
Alleles:				
A vs. G	3.612	0.0574	1.00	0.69–1.45
Genotypes:				
AA vs. GG	3.238	0.0720	1.96	0.94–4.07
GA vs. GG	3.637	0.0565	2.16	1.17–4.00
AA vs. GA	0.083	0.7735	1.10	0.57–2.13
*HCRTR1* *G29A				
Alleles:				
A vs. G	3.413	0.0647	2.21	0.94–5.22
Genotypes:				
GA vs. GG	3.607	0.0576	2.31	0.96–5.56

We also analyzed the clinical features of migraine in the presence of the S allele of 5-HTTLPRR and the A allele of *HCRTR1* G1222A polymorphisms. The average migraine attack frequency and the average duration of the headache between group S (SS and SL genotype) and group L (LL genotype) of 5-HTTLPR in MA and MO patients showed no statistical differences. Similarly, the results of the analysis of migraine frequency and length of the headache in group A (AA and GA) and group G (GG) of *HCRTR1* G1222A were negative.

### Biochemical Analyses

The significantly higher 5-HT plasma concentration was found in MA patients as compared to MO patients (*p* = 0.0308, Kruskal-Wallis test) or control subjects (*p* = 0.0369, Mann-Whitney test). No significant changes were observed in the HCRT-1 concentration (Table 4). The correlation between 5-HT and HCRT-1 occurred only in the control group (Spearman’s coefficient Rs = +0.4049, *p* = 0.010).

**Table 4:** 5-HT and HCRT-1 concentration in MA, MO, both migraine patients (MA+MO) and controls.

	Controls	MA	MO	Migraine patients	p(I)	p(II)
5-HT	0.095 ± 0.090;	0.123 ± 0.094^1,2^;	0.084 ± 0.086;	0.098 ± 0.091;	**0.0308**^1^	**0.0369**^2^
[μg/mL]	0.078;	0.0121;	0.068;	0.073;		0.3084
	0.029, 0.140	0.053, 0.170	0.018, 0.110	0.023, 0.151		0.7240
HCRT-1	664.1 ± 406.0;	648.1 ± 341.4;	715.9 ± 358.9;	684.4 ± 349.6;	0.7273	0.9900
[pg/mL]	542.10;	592.65;	678.75;	609.90;		0.4779
	319.8, 1007.1	329.35, 962.28	384.88, 1058.88	357.85, 1033.53		0.6405

*The values in each cell represent: mean ± standard deviation; median; quartile 1, and quartile 3. MA, migraine with aura, MO, migraine without aura, 5-HT, serotonin, HCRT-1, hypocretin-1. Kruskal-Wallis test (p I) and Mann-Whitney test (p II). Statistical significance at *p* < 0.05: ^1^between MA and MO (p I); ^2^between MA and controls (p II). Spearman’s coefficient, Rs = +0.4049, *p* = 0.010 between 5-HT and HCRT-1 in control group*.

We also correlated 5-HT and HCRT-1 concentrations with the clinical features of migraine. The MA and MO patients were divided into smaller groups according to the duration of migraine history, the average length of migraine attack and an average number of attacks per month. The 5-HT level was higher in patients with longer migraine history. The 5-HT concentration was increased in individuals suffering from MA between 5–10 years as compared to control and MO patients (*p* = 0.0212, Kruskal-Wallis test). The level of biochemical markers differed with the average length of the attacks. The lowest 5-HT concentration was observed in patients (both MA and MO) with attacks longer than 48 h. Higher HCRT-1 concentration was characterized by shorter migraine attacks, but this trend was observed only in the MO group. The increasing trend of 5-HT and HCRT-1 concentrations was observed in MA patients in the case of attack frequency, while in MO patients it was not.

### Genotype-Phenotype Correlations

To investigate the genotype-phenotype relationship, the plasma 5-HT and HCRT-1 levels were compared in individuals with different genotypes of 5-HTTLPR and *HCRTR1* G1222A, *G29A polymorphisms in the MA, MO, and control group (Tables 5, 6). The S allele of 5-HTTLPR tended to decrease the 5-HT concentration in the control group, while the opposite trend was observed in MA patients. The 5-HT level was statistically higher in MA subjects carrying SS 5-HTTLPR genotype than in control subjects with SS genotype (*p* = 0.0367, Mann-Whitney test, the power of test: 0.98). In the MO group, the 5-HT level was the lowest in patients with LL genotype. However, the HCRT-1 concentration was two-times lower in MA individuals carrying SS 5-HTTLPR than in controls and MO patients with this genotype.

**Table 5:** 5-HT concentration in MA, MO, both migraine patients (MA+MO) and control subjects regarding different genotypes of 5-HTTLPR, *HCRTR1* G1222A, and *G29A.

	Controls	MA	MO	Migraine patients	p (I)	p (II)
5-HTTLPR						
SS	0.057 ± 0.053	0.165 ± 0.035^1^	0.086 ± 0.104	0.111 ± 0.095	**0.0367**^1^	0.1519
SL	0.092 ± 0.098	0.122 ± 0.095	0.093 ± 0.076	0.106 ± 0.086	0.2049	0.1724
LL	0.106 ± 0.087	0.110 ± 0.104	0.072 ± 0.093	0.084 ± 0.097	0.0716	0.0951
*HCRTR1* G1222A						
GG	0.101 ± 0.102	0.140 ± 0.057^2^	0.074 ± 0.078	0.106 ± 0.075	**0.0375**^2^	0.4233
GA	0.094 ± 0.085	0.130 ± 0.109	0.083 ± 0.094	0.098 ± 0.100	0.1298	0.8161
AA	0.083 ± 0.077	0.093 ± 0.095	0.091 ± 0.075	0.092 ± 0.083	0.8636	0.6301
*HCRTR1* *G29A						
GG	0.095 ± 0.089	0.137 ± 0.094^3,4^	0.084 ± 0.089	0.103 ± 0.094	**0.0043**^3,4^	0.6078
GA	0.083 ± 0.111	0.062 ± 0.066	0.080 ± 0.064	0.070 ± 0.063	0.7965	0.8366

*The values in each cell represent: mean ± standard deviation. MA, migraine with aura, MO, migraine without aura. Kruskal-Wallis test (p I) and Mann-Whitney test (p II). Statistical significance at *p* < 0.05:^1^between MA and controls (p I); ^2^between MA and MO (p I); Statistical significance at *p* < 0.01: ^3^between MA and controls, ^4^between MA and MO (p I)*.

**Table 6:** HCRT-1 concentration in MA, MO, both migraine patients (MA, MO) and control subjects regarding different genotypes of 5-HTTLPR, *HCRTR1* G1222A and *G29A.

	Controls	MA	MO	Migraine patients	p (I)	p (II)
5-HTTLPR						
SS	668.8 ± 337.9	354.5 ± 161.7	740.3 ± 401.0	568.9 ± 362.7	0.1910	0.5360
SL	657.9 ± 450.6	734.4 ± 370.7	793.5 ± 335.6	764.8 ± 348.7	0.7080	0.4141
LL	668.0 ± 393.0	622.3 ± 277.4	572.0 ± 370.6	594.4 ± 324.1	0.8321	0.6935
*HCRTR1* G1222A
GG	752.5 ± 456.6	539.5 ± 332.7	471.2 ± 252.9	509.7 ± 293.0	0.3494	0.1551
GA	671.2 ± 389.2	715.7 ± 385.2	798.2 ± 365.0	769.1 ± 368.6	0.6266	0.4157
AA	451.5 ± 260.0	671.7 ± 278.3	683.4 ± 346.8	675.2 ± 280.0	0.2120	0.0845
*HCRTR1* *G29A
GG	662.6 ± 402.1	656.4 ± 327.4	723.1 ± 374.2	691.7 ± 351.0	0.7681	0.6129
GA	681.5 ± 502.9	598.3 ± 472.9	677.1 ± 293.8	642.1 ± 358.8	0.9463	>0.9999

*The values in each cell represent: mean ± standard deviation. MA, migraine with aura, MO, migraine without aura. Kruskal-Wallis test (p I) and Mann-Whitney test (p II)*.

The A allele of *HCRTR1* G1222A tended to decrease both 5-HT and HCRT-1 concentrations in the control group. In the migraine group (MA+MO) this decreasing trend was observed only in 5-HT levels. However, when we divided patients into MA and MO groups, the opposite relations occurred. In MA patients, the A allele of *HCRTR1* G1222A was associated with lower 5-HT levels, while in MO patients with higher 5-HT levels as compared to subjects with the G allele. The GA genotype of *HCRTR1* *G29A was associated with a lower 5-HT concentration than in individuals with GG genotype in all groups: controls, MA, MO. The 5-HT level was higher in MA patients with GG genotype than in control individuals with this genotype (*p* = 0.0043, Mann-Whitney test, the power of the test: 0.84). The GA genotype tended to increase the HCRT-1 concentration in the control individuals and to decrease in MA and MO patients.

## Discussion

According to our knowledge this is the first study investigating the correlation between serotonergic and hypocretinergic systems regarding genetic studies in migraine patients. The role of 5-HT in the migraine pathomechanism is undeniable, and migraine is described as a chronic low 5-HT syndrome. The reduced 5-HT and abnormal serotonergic neurotransmission may facilitate activation of CSD and TGVS (Hamel, 2007). Moreover, the serotonergic neurotransmission may be regulated by HCRT, hypothalamic neuropeptides that control pain perception and are involved in early phase of migraine attack (Holland and Goadsby, 2007). Taken together, evidences indicated the significant role of 5-HT and HCRT in migraine. The disturbances in serotonergic and hypocretinergic systems in migraine may be a consequence of polymorphisms in genes, such as *SLC6A4* and *HCRTR1*. For the genetic studies we chose 5-HTTLPR polymorphism of *SLC6A4* and G1222A, *G29A SNP of *HCRTR1*. We found no association between 5-HTTLPR and migraine. However, the *HCRTR1* G1222A seems to be a risk factor for MO, while *HCRTR1* *G29A for MA. The GA genotype of *G29A may contribute to the early age of onset of migraine.

The 5-HTTLPR is a widely studied deletion/insertion polymorphism in the *SLC6A4* gene and it occurs in two alleles: short (S) and long (L). The role of 5-HTTLPR was reported in the development of mental diseases, pain perception and mood disorders (Dorszewska et al., 2014). Its role in migraine predisposition is still unclear. The studies in the Asian population denied the association between S allele of 5-HTTLPR and migraine (Kotani et al., 2002; Kim et al., 2005; Park et al., 2006), while the studies in the European population are inconsistent. The association was found in Italian (Borroni et al., 2005), German (Marziniak et al., 2005), Hungarian (Juhasz et al., 2003a; Gonda, 2007) populations, while other studies in German, Austrian (Todt et al., 2006; Wieser et al., 2010), and Turkish (Yılmaz et al., 2001) populations indicated no association with migraine. We also found no differences in the genotype and allele frequencies of 5-HTTLPR in the Polish population. The divergences in European studies may result from different group sizes, characteristics of the examined group (female and/or male) and migraine subtypes (MA and/or MO). It needs to be noted that two meta-analyses of 5-HTTLPR were conducted in migraine patients and the results confirmed no association with this disease in the European and Asian populations (Schürks et al., 2010; Liu et al., 2011). However, Schürks et al. (2010) suggested that the S allele is a risk factor for migraine among European women (OR = 2.02; 95%CI 1.24–3.28), which underlines the mentioned ethnic differences.

A few research groups also correlated the *SLC6A4* 5-HTTLPR gene with the clinical course of migraine in different populations. In the Asian population, Kotani et al. (2002) linked SS genotype of 5-HTTLPR with more frequent migraine attacks than in patients with SL and LL. Whereas in the Polish population, we found no statistical differences in migraine frequency and headache duration between patients with S allele (SS and SL genotype) and without (LL genotype). These results correspond with a study performed by Wieser et al. (2010) which showed no association of 5-HTTLPR with attack frequency as well as no association with comorbid depression. It seems that 5-HTTLPR does not influence the clinical course of MA and MO.

MA and MO may be however, associated with polymorphisms in the *HCRTR1* gene. The *HCRTR1* G1222A polymorphism is a missense variant in exon 7 leading to the substitution of isoleucine by valine at position 408 (Ile408Val). This polymorphism is located in the cytoplasmic tail of the receptor, a possible binding site for G proteins. It is associated with increased phosphorylation potential and may change the signal transduction (Meerabux et al., 2005). The A allele of *HCRTR1* G1222A occurs in 37% of the European population, while it is more common in American (49%), African (72%) and East Asian (76%) populations. The *HCRTR1* *G29A is an SNP localized in 3′UTR region, and the A allele occurs in 7% of the European population (Ensembl Database, 2017). We demonstrated that the A allele of G1222A polymorphism was more frequent in the MO group (52%) when compared to the control group (42%), while the GA genotype of *G29A was more frequent in the MA patients (16%) than in the control group (7%). Statistical significances for both *HCRTR1* polymorphisms were not observed when we analyzed MA and MO patients together. It suggests that *HCRTR1* G1222A polymorphism may be a risk factor for MO, whereas *HCRTR1* *G29A for MA. Moreover, the study of Rainero et al. (2011b) pointed out that the AA genotype of *HCRTR1* G1222A is a risk factor for migraine (OR 1.81, 95% CI 1.03–3.19). When they analyzed MA and MO independently, they found this association only in patients suffering from MO. In our study, the A allele of this polymorphism approached the borderline of significance as a risk factor for migraine. We observed that the presence of the A allele of *HCRTR1* G1222A (AA and GA genotype) had no significant influence on migraine frequency and length of the headache as compared to the GG genotype. Moreover, analysis of Rainero et al. (2011b) showed no effects of *HCRTR1* G1222A on migraine clinical features (not only with frequency of migraine and duration of the attack, but also with nausea, vomiting, phono- and photophobia, pulsating pain, unilateral pain, and the age of onset of the disease).

On the other hand, we found an effect of *HCRTR1* *G29A at the age of onset of MA. Our results indicated that *HCRTR1* *G29A polymorphism might be associated with MA, which generally starts in the first two decades of life. All MA patients with the GA *G29A had a positive family history of migraine. MA female patients with the GA genotype of *HCRTR1* *G29A and the AA genotype of *HCRTR1* G1222A developed migraines at 30 years of life. This suggests that the AA genotype of *HCRTR1* G1222A eliminates the impact of the *G29A on MA early onset, but only in women.

It is known that HCRT can modulate 5-HT action through a direct (excitatory projections) and indirect (inhibitory projections) pathway. Another mechanism involves the activation of GABAergic and NE neurons by HCRT. GABAergic neurons inhibit, while the NE neurons activate the 5-HT neurons (Holland and Goadsby, 2007). The opposite mechanism of action of the HCRT depends on its level: low concentrations of HCRT-1 and HCRT-2 have the direct excitatory effect on the 5-HT neurons, while the indirect, inhibitory effect is characteristic for higher concentrations (Liu et al., 2002). The NE neurons can also excite GABAergic neurons, which can inhibit HCRT neurons. Moreover, the 5-HT inhibits the activity of HCRT neurons (Li et al., 2016). Therefore, we correlated the genotypes of the 5-HTTLPR, *HCRTR1* G1222A and *G29A with the plasma concentrations of 5-HT and HCRT-1.

We found a correlation in the control group: the S allele of 5-HTTLPR was associated with lower 5-HT concentration than the L allele. The cell line studies explained the impact of 5-HTTLPR on the 5-HT level. Heils et al. (2002) indicated that the S allele of 5-HTTLPR polymorphism is associated with the downregulation of gene expression and two times lower rate of 5-HTT transcription compared to the L allele. This leads to a decrease in 5-HT uptake, thus longer serotonergic activity and lower plasma 5-HT concentration. We showed a lack of correlation in migraine, perhaps due to the alteration in this mechanism in this disease. We observed a higher 5-HT concentration in MA patients with the SS genotype than in control subjects with this genotype. The higher 5-HT level in the presence of the SS genotype, as compared to the SL and LL, was also reported by the group of Juhasz et al. (2003b). They found an association of the SS genotype and high 5-HT concentration in the whole population (both migraine and controls), but their migraine group consisted of only MO patients.

We showed that changes in the 5-HT level might be associated with polymorphisms in *HCRTR1* gene, as well. The A allele of *HCRTR1* G1222A tended to decrease both 5-HT and HCRT-1 concentrations as compared to the G allele, but only in healthy subjects. Furthermore, we observed lower 5-HT levels in individuals with the GA genotype of *HCRTR1* *G29A than in individuals with the GG in all analyzed groups (both migraine and controls). The AA genotype of *HCRTR1* G1222A was previously linked by Rainero et al. (2011a) with a 2.5-fold increased risk for developing major mood disorders in comparison with the GG genotype, probably due to altered 5-HT levels. We found the decreasing trend of 5-HT concentration in the presence of the A allele of *HCRTR1* G1222A only in MA patients, while in MO patients the trend was increasing. No such trend was observed in HCRT-1 concentration. It is possible that polymorphisms in the *HCRTR1* gene may alter the level of 5-HT and indirectly HCRT-1.

We found a positive correlation between 5-HT and HCRT-1 level only in the control group. However, the 5-HT concentration was different in patients with various clinical features of the disease, such as duration of migraine history, the frequency of attacks and length of the headache. Although, plasma HCRT-1 concentration did not correlate with the duration of migraine history and attack frequency, both in MA and MO patients. The level of HCRT-1 was similar in MA and MO patients suffering from longer or shorter headaches. Peres et al. (2011) studied HCRT-1 levels in CSF of chronic migraine patients and did not notice significant differences as compared to controls. While Sarchielli et al. (2008) found elevated levels of HCRT-1 in CSF of a chronic migraine and medication-overuse headache patients.

The limitation of the study is a relatively small sample size, cross sectional study design, difficulties in identifying casual relations due to lack of functional studies of *HCRTR1* G1222A and *G29A. It is not guaranteed that younger people from the control group will not suffer from migraine in the future.

## Conclusion

In conclusion, both serotonergic and hypocretinergic systems are involved in the migraine pathomechanism. The 5-HTTLPR polymorphism influences 5-HT concentrations. It seems that G1222A and *G29A polymorphisms in the *HCRTR1* gene may be associated with different subtypes of migraine. The *HCRTR1* G1222A polymorphism (located in exon 7) may be a risk factor for MO, while the *HCRTR1* *G29A (located in 3′UTR) for MA. The strict relation between 5-HT and HCRT-1 may be altered in migraine patients due to different genetic variants of 5-HTTLPR and *HCRTR1* G1222A or *G29A. Both plasma concentration of 5-HT and localization of *HCRTR1* polymorphisms may influence the clinical presence of migraine. Further research is needed to expand on the clinical and biological knowledge of migraine, however, it seems that functional studies of analyzed polymorphisms, especially *HCRTR1* *G29A, may bring new information about its role in the pathomechanisms of migraine.

## Data Availability

Additional datasets are available on request. Requests to access the datasets should be directed to the corresponding author.

## Author Contributions

MK: analysis of molecular biology and ELISA, preparation of samples for HPLC/EC, evaluation of molecular tests, participation in manuscript editing. MK-P: diagnosis of patients with migraine, clinical evaluation of the results. TG: statistical evaluation of results. EW: analysis of molecular biology, evaluation of molecular tests. WK: participation in research design, diagnosis of patients with migraine, clinical evaluation of the results; JD: participation in research design, HPLC analysis of samples, coordination of research, substantive assessment of study results, participation in manuscript editing.

## Conflict of Interest Statement

The authors declare that the research was conducted in the absence of any commercial or financial relationships that could be construed as a potential conflict of interest.
